# Supplementary material for: High-Frequency Recombination of Human Adenovirus in Children with Acute Respiratory Tract Infections in Beijing, China
Source: Viruses. 2024 May 23;16(6):828. doi: 10.3390/v16060828 (PMC11209268; doi:10.3390/v16060828)
Supplement: Supplementary file 1 [file viruses-16-00828-s001.zip › Table S1.pdf]

Table S1 The reference genome sequences downloaded from GenBank

Country of origin	Year	Name	Length (bp)	Accession number
USA	1953	HAdV-1 prototype strain	36001	AF534906
USA	1953	HAdV-2 prototype strain	35937	NC_001405
USA	1953	HAdV-5 prototype strain	35938	AC_000008
USA	1953	HAdV-6 prototype strain	35758	FJ349096
RUS	2001	HAdV-57 prototype strain	35818	HQ003817
GER	2015	HAdV-89 prototype strain（29C2）	35752	MH121097
CHN	2017	HADV-104 prototype strain	35933	MH558113
JPN	1987	strain:870550	35698	LC068713
JPN	1993	strain:930113	35758	LC068714
JPN	1994	strain:940162	35776	LC068715
JPN	2003	strain:1030787	35,778	LC068716
JPN	2004	strain:1040264	35746	LC068717
JPN	2004	strain:1040502	35774	LC068718
JPN	2005	strain:1050158	35735	LC068720
JPN	1987	strain:870550	35779	LC068712
JPN	2005	strain:1050156	35745	LC068719
USA	1992	human/USA/Pitts 00109/1992/2[P2H2F2]	35690	KF268310
USA	2002	T215/Ft Jackson South Carolina USA/2002	35868	KX384959
USA	2003	human/USA/VT5544/2003/2[P2H2F2]	35923	JX173084
USA	2003	human/USA/VT384/2003/1[P1H1F1]	35993	JX173082
USA	2003	human/USA/VT2672/2003/1[P1H1F1]	36003	JX173083
USA	2003	human/USA/VT2612/2003/1[P1H1F1]	36006	JX173085
USA	2004	human/USA/UFL Adv2/2004/2[P2H2F2]	35931	KF268130
USA	2004	human/USA/VT13862/2004/1[P1H1F1]	36002	JX173086
USA	2008	human/USA/UFL Adv5/2008/5[P2/H5/F5]	35928	KF268199
USA	2005	human/USA/UFL_Adv6/2005/6[P6H6F6]	35979	KF268129
USA	2007	human/USA/ak31_AdV6/2007/6[P6H6F6]	35747	JX423389
USA	1988	human/USA/CL_42/1988/5[P5H5F5]	35931	KF268127
USA	1990	human/USA/Pitts_00149/1990/5[P5H5F5], strain F268310	35911	KF429754

ARG	2000	human/ARG/A15812/2000/1[P1H1F1]	35989	JX173078
ARG	2002	human/ARG/A15932/2002/2[P2H2F2]	35925	JX173079
ARG	2013	human/ARG/A8649/2005/2[P2H2F2]	35925	JX173077
CHN	2000	human/Shanxi-CHN/105/2000	35951	MK041241
CHN	2000	human/Shanxi-CHN/106/2000	35899	MK041242
CHN	2000	human/Shanxi-CHN/141/2000	35958	MK041231
CHN	2001	human/Shanxi-CHN/160/2001	35906	MK041230
CHN	2001	human/Shanxi-CHN/135/2001	35917	MK041235
CHN	2002	human/Shanxi-CHN/22/2002	35922	MK041234
CHN	2003	human/Shanxi-CHN/154/2003	31220	MK041232
CHN	2006	human/Shanxi-CHN/106/2010	36023	MK041227
CHN	2002	human/Shanxi-CHN/87/2002	35758	MK041239
CHN	2012	human/Shaanxi-CHN/12045/2012	35935	MK041238
CHN	2002	human/Shanxi-CHN/151/2002	35754	MK041240
CHN	2006	human/Shanxi-CHN/32/2006	35994	MK041244
CHN	2007	human/Shanxi-CHN/38/2007	35932	MK041237
CHN	2010	human/Hebei-CHN/6645/2010	35940	MK041245
CHN	2012	human/Hunan-CHN/E177/2012	36024	MK041246
CHN	2012	human/CHN/BJO4/2012/[P1/H2/F2]	35953	MF315028
CHN	2013	human/CHN/BJ09/2013/[P1/H2/F2]	35958	MF315029
CHN	2013	human/Yunnan-CHN/13/2013	35943	MK041247
CHN	2013	human/Changchun-CHN/255/2013	35935	MK041243
CHN	2013	human/Yunnan-CHN/5240/2013	35952	MK041248
CHN	2013	human/Changchun-CHN/114/2013	35814	MK041233
CHN	2014	human/Yunnan-CHN/472/2014	35894	MK041229
CHN	2015	human/Xizang-CHN/55/2015	35934	MK041236
CHN	2015	human/Xizang-CHN/150/2015	35932	MK041228
CHN	2015	human/Gansu-CHN/1111872/2015	36014	MK041225
CHN	2016	human/CHN/SH/2016/1[P1H1F1]	35946	MH183293
CHN	2018	strain QH-1665/2018	36014	MN737436
CN	2016	C1ONP04Cu1Aug2016	35987	OM112294
CN	2015	C1ONP02Cu1Apr2015	36002	OM112293
CN	2015	C1ONP01Pr1Feb2015	36019	OM112292
CN	2018	C1ONP05Pr1Jan2018	36007	OM112290

CN	2016	C2ONP02Cu1Jan2016	35914	OM112289
CN	2015	C2ONP05Cu1Apr2015	35922	OM112291
CN	2018	C2ONP06Cu1Jan2018	35911	OM112287
CN	2018	C2ONP07Pr1May2018	35920	OM112286
CN	2015	C2ONP01Cu1Nov2015	35933	OM112285
CN	2016	C2ONP03Pr1Oct2016	35941	OM112284
CN	2015	C1ONP03Cu1Jun2015	36017	OM112288
VN	2015	vzhadvc5	35928	MH828486
VN	2014	vzhadvc2	35940	MH828485
VN	2014	vzhadvc1-5	35940	MH828484
VN	2014	vzhadvc1-1	36003	MH828480
VN	2014	vzhadvc1-2	36003	MH828481
VN	2015	vzhadvc1-3	36003	MH828482
VN	2015	vzhadvc1-4	36003	MH828483
CHN	2017	GD4163	35883	MN088492
CHN	2004	SX-2004-327	35932	MK165453
CHN	2000	SX-2000-140	35949	MK165452
RUS	2021	Novosibirsk/8.135Hl/2021	36009	ON152653
RUS	2021	Novosibirsk/7.45Hl/2021	35920	ON152652
RUS	2021	Novosibirsk/7.17Hl/2021	35934	ON152651
RUS	2022	Novosibirsk/7.273Hp/2022	35915	ON152650
RUS	2022	Novosibirsk/7.2Hp/2022	35910	ON152649
RUS	2020	Novosibirsk/8.171V/2020	35937	MZ151865
RUS	2020	Novosibirsk/8.142V/2020	35953	MZ151864
RUS	2020	Novosibirsk/8.202V/2020	35936	MZ151863
RUS	2020	Novosibirsk/8.234V/2020	35931	MZ151862
RUS	2019	Novosibirsk/7.134V/2019	35947	MZ151861
BR	2015	245-Araguaina	36100	MN628614
CHN	2016	DT-4（P1H2F2)	35873	MK357715
EGY	2001	human/EGY/E53/2001/2[P2H2F2]	35928	JX173081
EGY	2001	human/EGY/E13/2001/1[P1H1F1]	35936	JX173080
GER	2002	4C1	35999	MH121073
GER	2008	6C1	35859	MH121075
GER	2009	8C2	35783	MH121077

GER	2009	9C2	35845	MH121078
GER	2010	10C2	35843	MH121079
GER	2012	11C2	35848	MH121080
GER	2012	12Cl	35893	MH121081
GER	2012	13C1	35840	MH121082
GER	2012	14C2	35784	MH121083
GER	2012	16C2	35702	MH121085
GER	2013	17C2	35747	MH121086
GER	2013	18C1	35594	MH121087
GER	2013	19C2	35962	MH121088
GER	2013	20C1	35978	MH121089
GER	2014	21C2	35819	MH121090
GER	2014	22C1	36001	MH121091
GER	2014	23C2	35929	MH121092
GER	2014	24C2	35911	MH121093
GER	2014	25C5	35897	MH121094
GER	2014	26C2	35779	MH121095
GER	2015	27C2	35828	MH121096
GER	2015	28C5（5var）	35805	MF681662
GER	2015	31C2	35799	MH121099
GER	2015	32Cl	35932	MH121100
GER	2015	33C2	35853	MH121101
GER	2015	35C2	35949	MH121103
GER	2016	41C1	36077	MH121108
GER	2017	42C2	35777	MH121109
GER	2017	43C1	35913	MH121110
GER	2017	44C2	35918	MH121111
GER	2017	45C6	35753	MH121112
GER	2017	47C2	35923	MH121114
GER	2017	50C1	35926	MH121116
GER	2017	51Cl	35831	MH121117
USA	2010	HAdV-C89/USA/86674/2010	35917	OQ518342
USA	2011	HAdV-C89/USA/85996/2011	35900	OQ518341
USA	2009	HAdV-C89/USA/69915/2009	35913	OQ518326

USA	2011	HAdV-C89/USA/66423/2011	35926	OQ518318
USA	2011	HAdV-C89/USA/61634/2011	35899	OQ518313
USA	2010	HAdV-C89/USA/52944/2010	35911	OQ518302
USA	2009	HAdV-C89/USA/43142/2009	35910	OQ518291
USA	2011	HAdV-C89/USA/00774/2011	35920	OQ518255
SG	2016	SG06/HAdvC2/2016	35924	MN513342
SG	2016	SG05/HAdvC1/2016	35981	MN513341
SG	2016	SG08/HAdvC1/2016	35986	MN513344
SG	2016	SG09/HAdvC1/2016	35981	MN513345
CHN	2014	HK91	35954	MF044052
USA	2013	HAdV-C2/USA/97313/2013	35922	OQ518352
USA	2010	HAdV-C2/USA/95676/2010	35920	OQ518350
USA	2009	HAdV-C2/USA/79986/2009	35931	OQ518339
USA	2011	HAdV-C2/USA/78764/2011	35920	OQ518337
USA	2010	HAdV-C2/USA/78511/2010	35918	OQ518335
USA	2011	HAdV-C2/USA/75525/2011	35941	OQ518330
USA	2009	HAdV-C2/USA/68102/2009	35930	OQ518325
USA	2010	HAdV-C2/USA/59985/2010	35920	OQ518312
USA	2012	HAdV-C2/USA/59611/2012	35,922	OQ518309
USA	2009	HAdV-C2/USA/42316/2009	35941	OQ518290
USA	2010	HAdV-C2/USA/37676/2010	35919	OQ518285
USA	2009	HAdV-C2/USA/24111/2009	35932	OQ518272
USA	2011	HAdV-C2/USA/22725/2011	35914	OQ518271
USA	2011	HAdV-C2/USA/08285/2011	35938	OQ518261
USA	2010	HAdV-C2/USA/00543/2010	35764	OQ518254
USA	2012	HAdV-C5/USA/98124/2012	35906	OQ518353
USA	2009	HAdV-C5/USA/80077/2009	35904	OQ518340
USA	2009	HAdV-C5/USA/66723/2009	35914	OQ518319
USA	2012	HAdV-C5/USA/57124/2012	35906	OQ518307
USA	2010	HAdV-C5/USA/56304/2010	35906	OQ518305
USA	2011	HAdV-C5/USA/56086/2011	35914	OQ518303
USA	2012	HAdV-C5/USA/50045/2012	35906	OQ518296
USA	2012	HAdV-C5/USA/41891/2012	35906	OQ518289
USA	2009	HAdV-C5/USA/34713/2009	35940	OQ518283

USA	2011	HAdV-C5/USA/28636/2011	35923	OQ518277
USA	2010	HAdV-C5/USA/27885/2010	35908	OQ518275
USA	2012	HAdV-C5/USA/26107/2012	35925	OQ518274
USA	2011	HAdV-C5/USA/18603/2011	35903	OQ518268
USA	2012	HAdV-C5/USA/10166/2012	35906	OQ518263
CHN	2016	SH2016	35946	MH183293
USA	2012	HAdV-C1/USA/92827/2012	35998	OQ518349
USA	2013	HAdV-C1/USA/91767/2013	36002	OQ518347
USA	2013	HAdV-C1/USA/91094/2013	36002	OQ518346
USA	2012	HAdV-C1/USA/79813/2012	35987	OQ518338
USA	2009	HAdV-C1/USA/59915/2009	36001	OQ518311
USA	2011	HAdV-C1/USA/59814/2011	35977	OQ518310
USA	2013	HAdV-C1/USA/52647/2013	36002	OQ518301
USA	2009	HAdV-C1/USA/52197/2009	35993	OQ518300
USA	2009	HAdV-C1/USA/31835/2009	35996	OQ518280
USA	1968	HAdV-C1/USA/22397/1968	35998	OQ518270
USA	2010	HAdV-C1/USA/21605/2010	35988	OQ518269
USA	2009	HAdV-C1/USA/08707/2009	36007	OQ518262
USA	2011	HAdV-C1/USA/04787/2011	35979	OQ518259
