# Supplementary material for: High-Frequency Recombination of Human Adenovirus in Children with Acute Respiratory Tract Infections in Beijing, China
Source: Viruses. 2024 May 23;16(6):828. doi: 10.3390/v16060828 (PMC11209268; doi:10.3390/v16060828)
Supplement: Supplementary file 1 [file viruses-16-00828-s001.zip › Table S2.pdf]

Table S2 The closest match genes and their nucleotide identity (%)

Genome seqences	the closest match penton base gene (identity %)	the closest match hexon gene (identity %)	the closest match fiber gene (identity %)
CHN-BJ-86413/2017	LC504573(99.76%)	LC504573(99.94%)	MN628615(100%)
CHN-BJ93578/2018	LC504573(99.59%)	KF268199(99.81%)	KF268199(100%)
CHN-BJ-95031/2018	KF268129(99.20%)	KF268127(99.81%)	KF429754(99.54%)
CHN-BJ-1w5060/2019	MZ151865(100%)	MK883607(100%)	MK883607(100%)
CHN-BJ-S8130/	AB448767（99.75%）	EF121005（99.94%）	AB448767（100%）
