# Supplementary material for: High-Frequency Recombination of Human Adenovirus in Children with Acute Respiratory Tract Infections in Beijing, China
Source: Viruses. 2024 May 23;16(6):828. doi: 10.3390/v16060828 (PMC11209268; doi:10.3390/v16060828)
Supplement: Supplementary file 1 [file viruses-16-00828-s001.zip › Table S3.pdf]

Table S3 Possible recombination events of four HAdV-C and one HAdV-D strains identifed by RDP4.

Recombinant Strain	Parent Major/Minor	Recombinant Region in Alignment	Model (Average *P* Value)						
			RDP	GENECONV	Bootscan	Maxchi	Chimaera	SiSscan	3Seq
CHN-BJ-86413/2017	AC_000008/MH558113	15010-28171	2.686×10^{2}	1.102×10^{2}	3.592×10^{3}	6.455×10^{4}	5.182×10^{4}	7.587×10^{12}	9.690×10^{5}
CHN-BJ-95031/2018	AF534906/AC_000008	27794-31362	9.320×10^{17}	1.450×10^{53}	3.186×10^{75}	1.269×10^{38}	1.118×10^{25}	4.251×10^{89}	4.389×10^{11}
CHN-BJ93578/2018	AC_000008/HQ003817	12036-14847	4.584×10^{20}	2.483×10^{16}	3.042×10^{13}	9.284×10^{16}	9.540×10^{5}	/	3.854×10^{13}
CHN-BJ-1W5060/2019	NC_001405/AF534906	11814-19207	5.214×10^{53}	1.317×10^{51}	7.691×10^{22}	2.690×10^{20}	3.340×10^{19}	3.403×10^{18}	1.865×10^{14}
CHN-BJ-S8130/2021	FJ169625 /LC314153	11,444-17,721	2.708×10^{121}	1.520×10^{127}	4.229×10^{124}	5.996×10^{35}	1.558×10^{35}	2.197×10^{39}	2.220×10^{15}
		17,722-20,998	3.401×10^{187}	9.744×10^{187}	2.256×10^{190}	6.439×10^{39}	9.486×10^{40}	8.145×10^{42}	6.661×10^{15}
