# Supplementary figures and images for: High-Frequency Recombination of Human Adenovirus in Children with Acute Respiratory Tract Infections in Beijing, China
Source: Viruses. 2024 May 23;16(6):828. doi: 10.3390/v16060828 (PMC11209268; doi:10.3390/v16060828)

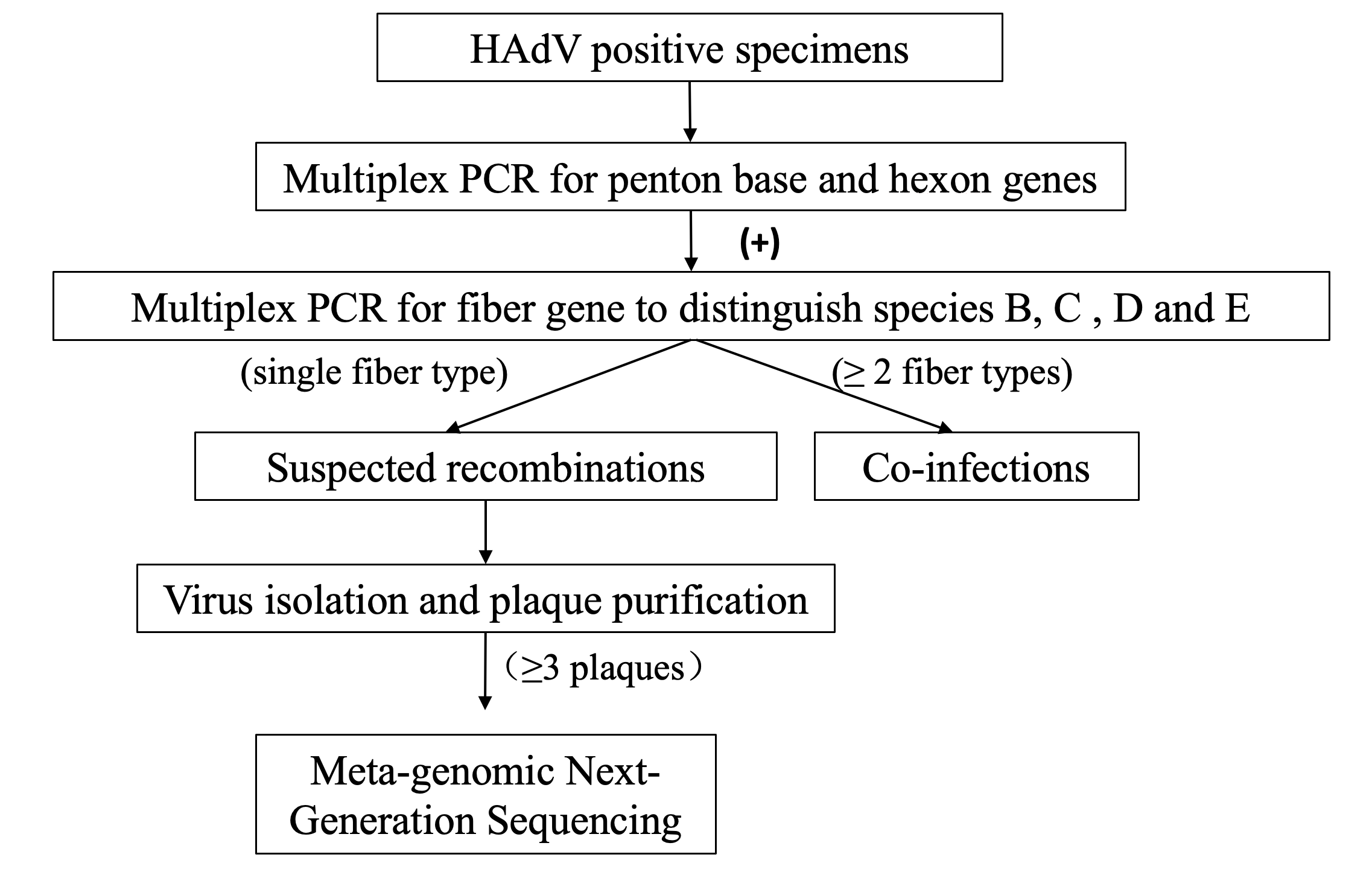

Supplement: Supplementary file 1 [file viruses-16-00828-s001.zip › Figure S1.png]
